# PaOctβ2R: Identification and Functional Characterization of an Octopamine Receptor Activating Adenylyl Cyclase Activity in the American Cockroach *Periplaneta americana*

**DOI:** 10.3390/ijms23031677

**Published:** 2022-01-31

**Authors:** Wolfgang Blenau, Anna-Sophie Bremer, Yannik Schwietz, Daniel Friedrich, Lapo Ragionieri, Reinhard Predel, Sabine Balfanz, Arnd Baumann

**Affiliations:** 1 Institute of Biochemistry, Leipzig University, 04103 Leipzig, Germany; wolfgang.blenau@uni-leipzig.de; 2 Institute of Biological Information Processing, IBI-1, Research Center Jülich, 52428 Jülich, Germany; s0anbrem@uni-bonn.de (A.-S.B.); s.balfanz@fz-juelich.de (S.B.); 3 Department of Biology, Philipps University of Marburg, 35037 Marburg, Germany; yannikschwietz@icloud.com; 4 Department of Molecular and Cellular Biology, Harvard University, Cambridge, MA 02138, USA; daniel_friedrich@fas.harvard.edu; 5 Institute of Zoology, University of Cologne, 50923 Cologne, Germany; lapo.ragionieri@uni-koeln.de (L.R.); reinhard.predel@uni-koeln.de (R.P.)

**Keywords:** biogenic amines, cellular signaling, cockroach, gene annotation, gene family, GPCR, second messenger

## Abstract

Biogenic amines constitute an important group of neuroactive substances that control and modulate various neural circuits. These small organic compounds engage members of the guanine nucleotide-binding protein coupled receptor (GPCR) superfamily to evoke specific cellular responses. In addition to dopamine- and 5-hydroxytryptamine (serotonin) receptors, arthropods express receptors that are activated exclusively by tyramine and octopamine. These phenolamines functionally substitute the noradrenergic system of vertebrates Octopamine receptors that are the focus of this study are classified as either α- or β-adrenergic-like. Knowledge on these receptors is scarce for the American cockroach (*Periplaneta americana*). So far, only an α–adrenergic-like octopamine receptor that primarily causes Ca^2+^ release from intracellular stores has been studied from the cockroach (PaOctα1R). Here we succeeded in cloning a gene from cockroach brain tissue that encodes a β-adrenergic-like receptor and leads to cAMP production upon activation. Notably, the receptor is 100-fold more selective for octopamine than for tyramine. A series of synthetic antagonists selectively block receptor activity with epinastine being the most potent. Bioinformatics allowed us to identify a total of 19 receptor sequences that build the framework of the biogenic amine receptor clade in the American cockroach. Phylogenetic analyses using these sequences and receptor sequences from model organisms showed that the newly cloned gene is an β2-adrenergic-like octopamine receptor. The functional characterization of PaOctβ2R and the bioinformatics data uncovered that the monoaminergic receptor family in the hemimetabolic *P. americana* is similarly complex as in holometabolic model insects like *Drosophila melanogaster* and the honeybee, *Apis mellifera*. Thus, investigating these receptors in detail may contribute to a better understanding of monoaminergic signaling in insect behavior and physiology.

## 1. Introduction

Biogenic amines play a pivotal role in the regulation of physiology and behavior of most animals. In insects and other protostomes, tyramine and octopamine functionally substitute the noradrenergic/adrenergic system present in most vertebrates. The two phenolamines can act as neurotransmitters, neuromodulators and neurohormones as well (for recent reviews, see: [1,2,3,4]). Besides the vinegar fly *Drosophila melanogaster* and the honeybee (*Apis mellifera*) as well-established model organisms for investigating the roles of biogenic amines on behavioral plasticity and social behavior [5,6,7,8,9], the cockroach (*Periplaneta americana*) is well suited as a model to examining the contribution of biogenic amines on physiology and neurobiology as well [10,11,12].

Both tyramine and octopamine bind to membrane proteins that belong to the superfamily of G protein-coupled receptors (GPCRs). There is increasing evidence that each phenolamine can activate several receptor subtypes that couple to distinct, yet receptor-subtype specific intracellular signaling pathways. In each of the insect model species, *D. melanogaster* and *A. mellifera*, at least two different tyramine and six different octopamine receptors were identified and characterized. Type 1 tyramine receptors (DmTAR1 [13], AmTAR1 [14,15]) inhibit adenylyl cyclase activity and cause a reduction in the intracellular cAMP concentration ([cAMP]_i_). DmTAR2 shows a high specificity for tyramine and likely leads to an increase in the intracellular Ca^2+^ concentration ([Ca^2+^]_i_; [16,17]). In contrast, the type 2 tyramine receptor from the honeybee, AmTAR2, was found to induce cAMP production upon activation [18]. A third tyramine receptor in *D. melanogaster*, DmTAR3 [16], is almost equally well activated by tyramine and phenylethylamine, and activation leads to both a decrease in [cAMP]_i_. and an increase [Ca^2+^]_i_ [17]. Octopamine receptors can be differentiated in α–adrenergic-like and β-adrenergic-like receptors [19]. The α–adrenergic-like receptors evoke Ca^2+^ release from intracellular stores (DmOctα1A/BR [20,21], AmOctα1R [22]) or inhibit adenylyl cyclases (DmOctα2R [23], AmOctα2R [24]). The β-adrenergic-like receptors stimulate adenylyl cyclases and thereby raise [cAMP]_i_ (DmOctβ1-3R [24,25], AmOctβ1-4R [26]). Cockroaches also share a long tradition as model organisms to perform basic research in physiology and neurobiology. Knowledge about the molecular and pharmacological properties of monoaminergic receptors in *P. americana*, however, is still sparse. So far, only two dopamine (PaDOP2A and PaDOP2B [27]) and one 5-hydroxytryptamine (serotonin) receptor (Pa5-HT1 [28]), two tyramine (PaTAR1A [29], PaTAR1B [30]) and one octopamine receptor (PaOctα1R [31]) have been functionally characterized.

Here we applied a molecular cloning and an in-silico approach to gain deeper insight into the monoaminergic receptor clade of *P. americana*. We succeeded in cloning a β-adrenergic-like octopamine receptor gene expressed in *P. americana* brain tissue. When constitutively expressed in a cell line, PaOctβ2R stimulated adenylyl cyclase activity. Notably, octopamine was two orders of magnitude more potent than tyramine in terms of activating the receptor. For both agonists the rank order of synthetic antagonists was similar, with epinastine being the most efficacious inhibitor. In addition to this functional characterization, we examined transcriptome data for the presence of monoaminergic receptors. Together with hitherto cloned receptor genes, a total of four dopamine, four serotonin, five octopamine, three tyramine receptors, and two putative biogenic amine receptors with unknown ligand specificity were identified. Thus, the monoaminergic receptor clade in *P. americana* is similarly complex as in other arthropods. We are convinced that these findings will significantly improve further physiological, neurobiological as well as pharmacological studies that rely on specific signaling of the monoaminergic system to modulate cockroach behavior.

## 2. Results

### 2.1. Molecular and Structural Properties of the PaOctβ2R

Based on currently known biogenic amine receptors from *A. mellifera* and *D. melanogaster*, degenerated primers were designed. We succeeded in cloning the complete cDNA encoding a PaOctβ2R by PCR on single stranded cDNA synthesized on mRNA isolated from adult cockroach brain tissue. The cDNA contains an open reading frame (ORF) of 1371 bp.

**Figure 1:**
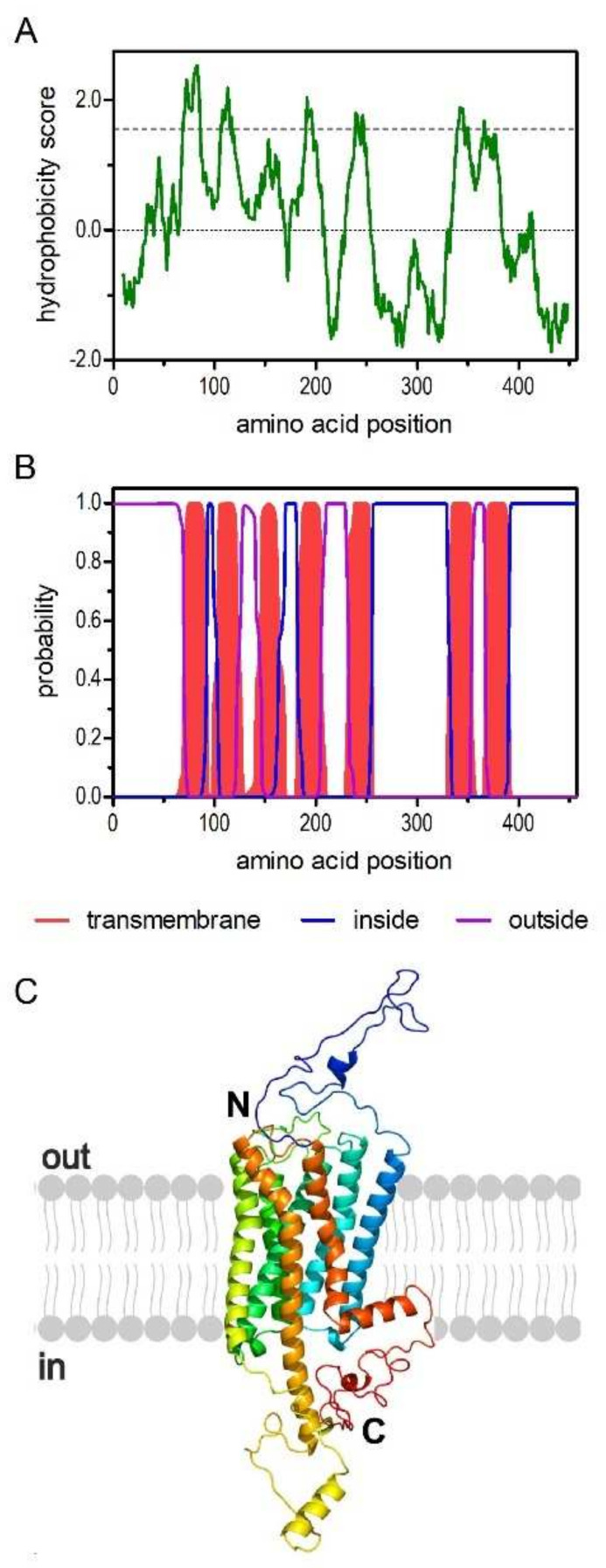
Structural characteristics of the amino acid sequence deduced for PaOctβ2R. (**A**) Hydrophobicity profile of PaOctβ2R. The profile was calculated according to Kyte and Doolittle algorithm [32] using a window size of 19 amino acids. Peaks with scores greater than 1.6 (dashed line) indicate possible transmembrane regions. (**B**) Prediction of transmembrane domains with TMHMM server v. 2.0 [33]. Putative transmembrane domains are indicated in red. Extracellular regions are shown with a purple line, intracellular regions with a blue line. (**C**) Color-coded (rainbow) 3D model of the receptor as predicted by Phyre2 [34]. The extracellular N-terminus (N) and the intracellular C-terminus (C) are labeled.

The deduced amino acid sequence consists of 457 residues with a calculated molecular weight of 52.1 kDa and a pI of 8.73. The hydrophobicity profile according to Kyte and Doolittle [32] and prediction of transmembrane helices using TMHMM Server v.2.0 [33] suggest seven transmembrane (TM) domains (Figure 1A,B), a cognate feature of GPCRs. The TM segments are flanked by an extracellular N-terminus of 69 residues and an intracellular C-terminus of 67 residues. We submitted the PaOctβ2R sequence to Phyre2 [34] and obtained a three-dimensional model of the receptor that supported the typical membrane arrangement of a GPCR (Figure 1C).

The primary sequence of the PaOctβ2R contains several putative sites for posttranslational modification (Supplementary Figure S1). Two potential N-glycosylation sites (N-X-(S/T)) are present in the extracellular N-terminus: N_21_ST and N_45_LT. Nine consensus sites for phosphorylation by protein kinase A and eight consensus sites for phosphorylation by protein kinase C are found in the cytoplasmic domains of the receptor protein (Figure S1). Cysteine residues (C_404_, C_407_, and C_410_) in the C-terminal loop might serve as palmitoylation sites. Insertion of these fatty acids into the plasma membrane additionally stabilizes the protein and might facilitate interaction with the corresponding G-protein.

In addition to these sites, several characteristic sequence motifs of GPCRs were identified in the primary structure of PaOctβ2R. The D_163_RY motif (D^3.49^R^3.50^Y^3.51^; labeling according to [35]) is located at the cytoplasmic end of TM3 (Figure S1). In TM7, the residues N_383_PLIY (N^7.49^P^7.50^L^7.51^I^7.52^Y^7.53^) constitute part of the hydrophobic interaction site with the phenyl moiety of the biogenic amine. Furthermore, D_146_ (D^3.32^) in TM3 and S_235/239_ (S^5.42/5.46^) in TM5 (Figure S1) most likely interact with the protonated amino group and hydroxyl group of the biogenic amine, respectively, as has been experimentally demonstrated for several members of biogenic amine receptor family [36,37].

**Figure 2:**
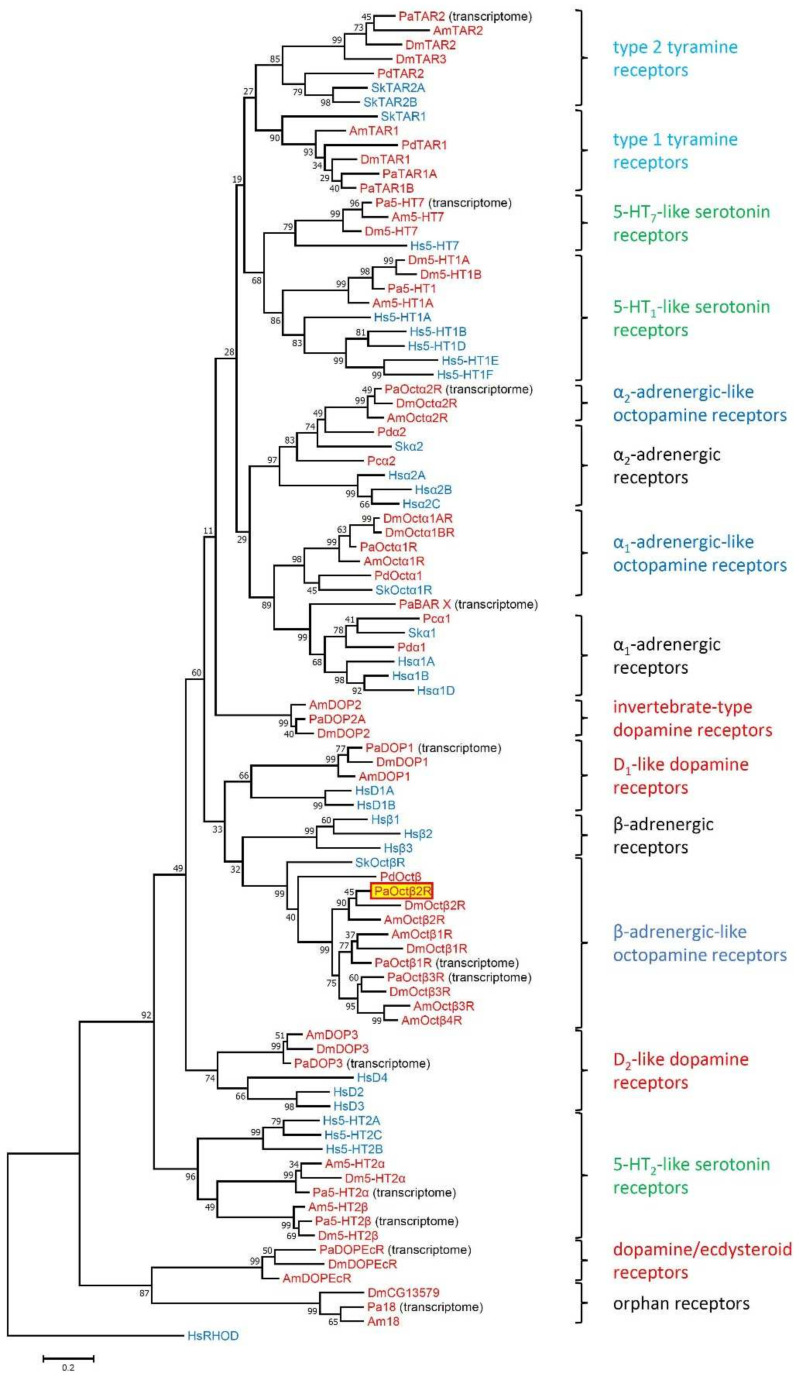
Phylogenetic relationships of monoaminergic receptors. Alignments were performed with BioEdit [40] by using the core amino-acid sequences of TM 1-4, TM 5, TM 6, and TM 7. The evolutionary history was inferred by using the Maximum Likelihood method based on the Poisson correction model. The tree with the highest log likelihood (−28600.17) is shown. The percentage of trees in which the associated taxa clustered together is shown next to the branches. The tree is drawn to scale, with branch lengths measured in the number of substitutions per site. The analysis involved 93 amino acid sequences. Human rhodopsin (HsRHOD) was used to root the tree. Receptor subclasses are given on the right. Abbreviations of species in alphabetical order are: Am *Apis mellifera*, Dm *Drosophila melanogaster*, Hs *Homo sapiens*, Pa *Periplaneta americana*, Pc *Priapulus caudatus*, Pd *Platynereis dumerilii*, Sk *Saccoglossus kowalevskii*. Protostomian species names are highlighted in red, whereas deuterostomian species names are given in blue. Accession numbers and annotations of all sequences used in the phylogenetic analysis can be found in Supplementary Table S1.

Several biogenic amine receptors have been physically cloned from cockroach tissue in recent years [27,28,29,30,31]. Lately, we screened for additional receptors of this gene family using transcriptome data derived from various tissues from *P. americana*. For that we used biogenic amine receptor sequences that have been functionally characterized previously in insect model organisms, specifically *D. melanogaster* and *A. mellifera.* A total of five potential dopamine (Dop1, 2A, 2B, 3, DopEcR), four 5-hydroxytryptamine (5-HT, serotonin; 5-HT1A, 2A, 2B, 7), five octopamine (Octα1R, α2R, β1R, β2R, β3R) and three tyramine (TAR1A, 1B, 2) receptors were identified. In addition, we identified two orphan receptors, PaBAR X (biogenic amine receptor X) and Pa18 (named after Am18, the orthologous receptor from the honeybee [38]). In order to gain insight into the phylogenetic relationship of the newly cloned PaOctβ2R receptor, multiple alignments were performed with these cockroach receptor sequences and biogenic amine receptors from protostomian and deuterostomian species using MEGA7 software (Figure 2; Table S1).

### 2.2. Annotation of P. americana Biogenic Amine Receptors and Phylogenetic Analysis of PaOctβ2R

Not all receptors binding to a certain biogenic amine compose uniform clusters in phylogenetic analyses, but the appropriate receptor subgroups do. PaOctβ2R assembled in a clade that contains the β-adrenergic-like octopamine receptor from *D. melanogaster* (DmOctβ1-3R [24,25])*, A. mellifera,* (AmOctβ1-4R [26], *Platynereis dumerilii* (PdOctβR [39]), and *Saccoglossus kowalevskii* (SkOctβR [39]). This clade is closely related to human β-adrenergic receptors. In contrast, α_1_-adrenergic-like octopamine receptors including PaOctα1R [31] are clearly set apart and form a sister group with α_1_-adrenergic receptors (Figure 2). The α_2_-adrenergic-like octopamine receptors form another separate branch and a sister group with the α_2_-adrenergic receptors (Figure 2).

The complete primary structures of the PaOctβ2R and PaOctα1R [31] receptors are only 18.6% identical and 30.5% similar. Notably, PaOctβ2R is more closely related to β_2_-adrenergic-like octopamine receptors from the drywood termite *Cryptotermes secundus* (89.1%/94.8.1%), the honeybee *A. mellifera* (69.1%/80.0%), and *D. melanogaster* (51.5%/63.0%). From the phylogenetic analyses we concluded that the newly cloned receptor most likely is a β2-adrenergic-like octopamine receptor.

### 2.3. Tissue Distribution of PaOctβ2R mRNA

**Figure 3:**
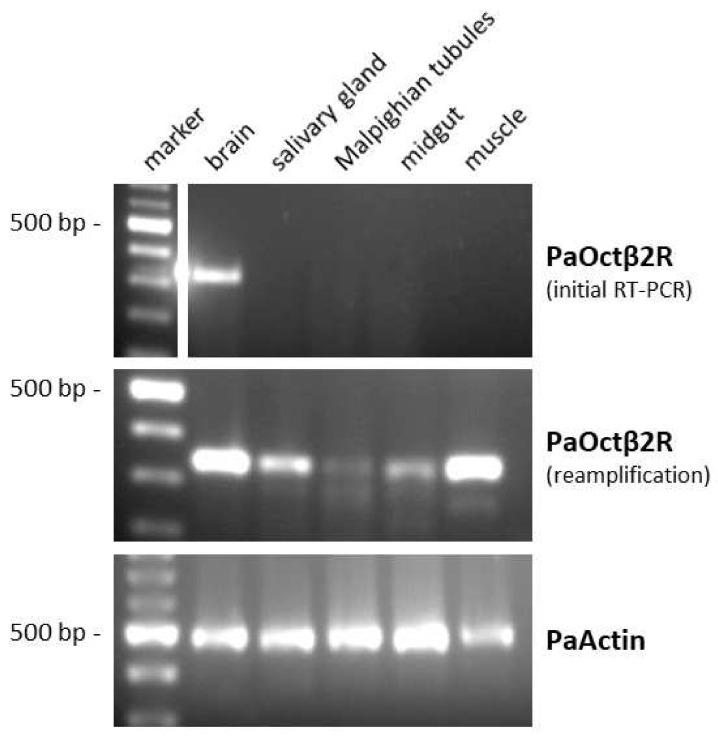
Tissue distribution of *PaOctβ2R* mRNA. A 100 bp DNA ladder is shown on the left. Detection of PCR products amplified on total RNA isolated from brain, salivary glands, Malpighian tubules, midgut, and leg muscle. Amplification failed when samples were digested with an RNAse cocktail prior to RT-PCR (data not shown). The lower panel shows RT-PCR products amplified with actin-specific primers (Accession No. AY116670) as a control.

The expression pattern of *PaOctβ2R* mRNA in various tissues of *P. americana* was investigated by RT-PCR with specific primers. The level of expression was generally very low. After the initial RT-PCR reaction, the *PaOctβ2R* transcript was only detected in the brain sample. After reamplification, the *PaOctβ2R* transcript could also be detected in samples of the leg muscle, the salivary gland, and the midgut (Figure 3). Receptor mRNA expression could not be reliably detected in samples from the Malpighian tubules. To ensure that the fragments were not amplified from genomic DNA, samples were treated with DNase I. When tissue samples were treated additionally with an RNase cocktail prior to initial RT-PCR, no PCR product could be amplified (data not shown).

Additional candidates for biogenic amine receptors, including two additional β-adrenergic-like octopamine receptors and two α-adrenergic-like octopamine receptors, were identified by screening the transcriptome data of various cockroach tissues. Table S2 summarizes the distribution of PaOctβ2R and all other monoaminergic receptors of the cockroach in the available transcriptomes.

### 2.4. Expression of PaOctβ2R-HA in flpTM Cells

**Figure 4:**
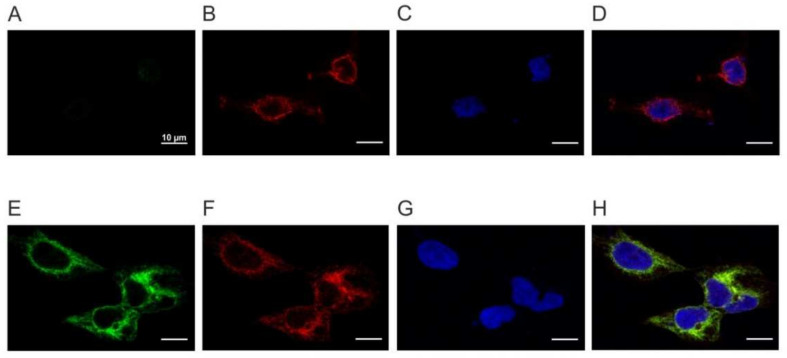
Confocal microscopy of flpTM and PaOctβ2R-HA-expressing flpTM cells. FlpTM (**A**–**D**) and PaOctβ2R-HA-expressing flpTM cells (**E**–**H**) were co-immunostained with rat anti-HA antibodies and specific antibodies against the CNG channel. (**A**,**E**) Samples were incubated with primary rat anti-HA antibodies (dilution 1:100) and secondary goat anti-rat-Alexa488 (dilution 1:500) antibodies. Non-transfected flpTM cells do not show fluorescent signals (**A**). In PaOctβ2R-expressing flpTM cells (**E**), the PaOctβ2R-HA protein was detected. (**B**,**F**) The same samples were incubated with specific antibodies directed against the C-terminus of the CNG channel (dilution 1:200) and secondary donkey anti-mouse-Cy3 (dilution 1:400) antibodies. In both cell lines, the CNG channel was detected. (**C**,**G**) Nuclei were stained with TOPRO-3 and are clearly differentiated from the cytosol. (**D**,**H**) Composite images.

To unravel the ligand specificity and intracellular signaling pathway activated by PaOctβ2R and to determine its pharmacological properties, flpTM cells were stably transfected with a construct C-terminally modified with an haemagglutinin A tag (PaOctβ2R-HA). Independent cell lines were obtained and examined by immuno-fluorescence staining for homogeneity (Figure 4) and Western blotting (Figure S2). Immunological staining of cells was performed with an anti-HA antibody detecting the tagged receptor (Figure 4, green). Since the cells also constitutively express a cyclic nucleotide-gated (CNG) channel, this protein was labeled with a specific antibody raised against the intracellular C-terminus (Figure 4, red). Cell nuclei were stained with TOPRO-3 (blue). The CNG channel but not the receptor protein was identified in flpTM cells that served as negative control (Figure 4A,B). In contrast, both proteins were stained in flpTM + PaOctβ2R-HA expressing cells (Figure 4E,F). Labeling was detected in the plasma membrane and in intracellular compartments.

To assess whether the receptor protein was post-translationally glycosylated, membrane proteins isolated from the receptor-expressing and parental (flpTM) cell lines were both treated with or without PNGase F and then separated by SDS-PAGE. Incubation of the Western blot membrane with anti-HA antibodies labeled two bands of ~68 and 60 kDa (Figure S2A, lane 1) in the sample that was not treated with PNGase F. Treating the proteins with PNGase F resulted in a complete loss of the 68 kDa band and an increase in the amount of the 60 kDa band (Figure S2A, lane 2). The apparent molecular weight corresponds well to the calculated value of PaOctβ2R-HA (53.4 kDa). No specific bands were labeled in lanes containing proteins from flpTM cells (Figure S2A, lanes 3 + 4). The blot was subsequently developed with an anti-CNG channel antibody (Figure S2B, lanes 1–4). In each lane, a single band was detected. Treatment with PNGase F resulted in a reduction of the apparent molecular weight of the channel protein (Figure S2B, lanes 2 + 4). Based on these results it is reasonable to assume that PaOctβ2R-HA is substantially glycosylated, at least in the heterologous expression system.

### 2.5. Ligand Specificity of the PaOctβ2R-HA Receptor

**Figure 5:**
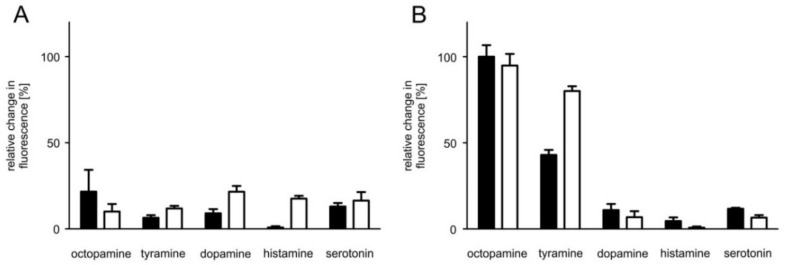
Biogenic amine evoked responses in flpTM and PaOctβ2R-HA-expressing cells. The relative change in fluorescence (corresponding to the amount of cAMP produced) in (**A**,**B**) is given as the percentage of the value obtained in the presence of 0.1 µM octopamine in flpTM + PaOctβ2R-HA cells (=100%). All measurements were performed in the presence of 100 µM 3-isobutyl-1-methylxanthine (IBMX). Biogenic amines were applied at two concentrations (0.1 µM (black bar) and 1 µM (white bar)). (**A**) Control measurements performed on flpTM cells did not result in increases in [cAMP]_i_. (**B**) Only octopamine and tyramine evoked an increase in [cAMP]_i_ in cells expressing PaOctβ2R-HA. A representative of four independent measurements is shown. Mean values ± SD of four-fold determinations is displayed.

The β-adrenergic-like octopamine receptors from, e.g., *D. melanogaster* [24,25] and *A. mellifera* [26] have been shown to stimulate adenylyl cyclase activity and thereby raise [cAMP]_i_ upon activation. These insect receptors are preferentially activated by octopamine and to a certain extent by tyramine as well. In a first series of experiments, we thus examined the ability of different biogenic amines, i.e., octopamine, tyramine, dopamine, histamine, and serotonin, to evoke PaOctβ2R-HA-dependent cAMP production in receptor-expressing as well as in the parental (flpTM) cell line. Increasing [cAMP]_i_ cause opening of the CNG channels and influx of extracellular Ca^2+^ which was detected by the Ca^2+^-sensitive dye FLUO-4. No stimulation of adenylyl cyclase activity was recorded in flpTM cells when incubated with 0.1 or 1 µM concentrations of biogenic amines (Figure 5A). In contrast, PaOctβ2R-HA-expressing cells showed increasing [cAMP]_i_ after application of both octopamine and tyramine. Neither dopamine, histamine nor serotonin caused an increase in [cAMP]_i_ (Figure 5B).

**Figure 6:**
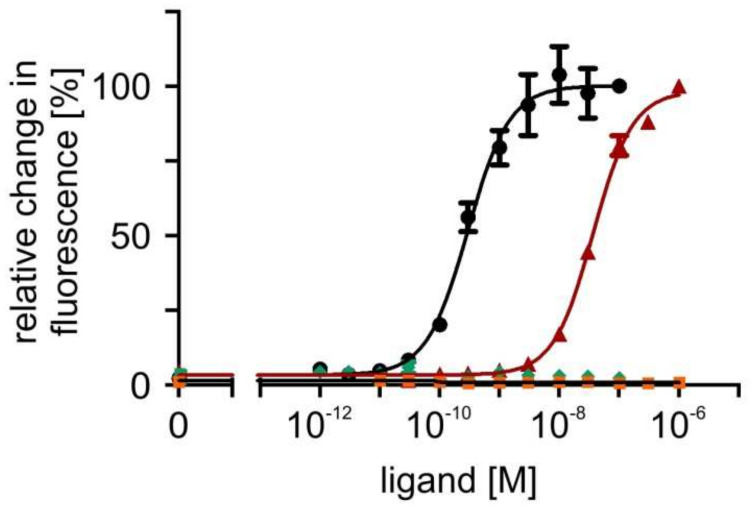
Concentration-dependent effects of octopamine and tyramine on [cAMP]_i_ in PaOctβ2R-HA-expressing and flpTM (control) cells. Relative change in fluorescence (corresponding to the amount of cAMP) is given as the percentage of the value obtained with the highest octopamine or tyramine concentration (=100%). All measurements were performed in the presence of 100 µM IBMX. Octopamine (•) and tyramine (▲) activation of PaOctβ2R-HA led to a concentration-dependent increase in the fluorescence signal. No change in the fluorescence signal was observed in flpTM cells (octopamine ♦; tyramine ■). A total of five independent measurements were performed. Data points represent the mean ± SD of a representative eight-fold determination.

In a next step, concentration series of octopamine and tyramine ranging from 10^−12^ M to 10^−7^ M for octopamine and 10^−11^ M to 10^−6^ M for tyramine were applied to the cell lines. The fluorescence signals were normalized to values obtained in flpTM + PaOctβ2R-HA cells with the highest ligand concentration (=100%) and used to calculate concentration response curves for the receptor (Figure 6; Table 1). The concentration-response curves for both phenolamines were sigmoid and saturated at an octopamine concentration of ≥10^−9^ M and a tyramine concentration of ≥10^−7^ M, respectively (Figure 6).

The ligand concentration leading to half maximal activation of PaOctβ2R-HA (EC_50_) was 2.97 × 10^−10^ M octopamine and 3.68 × 10^−8^ M tyramine, respectively (for mean values of all experiments, see Table 1). In non-transfected flpTM cells, no change in the fluorescence signal was observed upon application of either octopamine or tyramine. Accordingly, all subsequent measurements with antagonists (see Section 2.6) were carried out on an octopamine or tyramine background causing ~75% of the maximal response.

The pharmacological experiments conducted so far suggested that PaOctβ2R-HA has a clear (~100 fold) preference for octopamine over tyramine and can therefore be considered a functional β-adrenergic-like octopamine receptor.

### 2.6. Pharmacological Properties of the PaOctβ2R-HA Receptor

**Figure 7:**
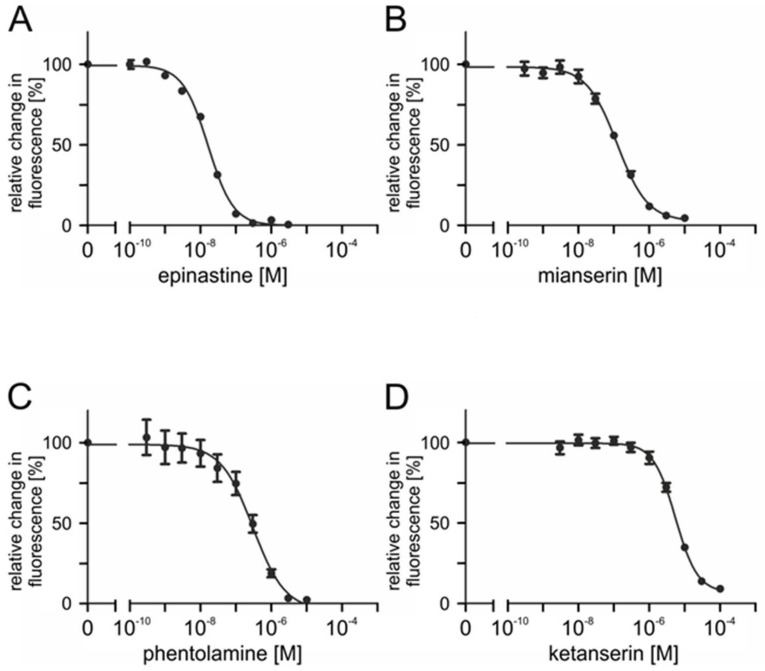
Effects of putative antagonists on octopamine-activated PaOctβ2R-HA. Concentration series of (**A**) epinastine, (**B**) mianserin, (**C**) phentolamine, and (**D**) ketanserin were applied in the presence of 1 × 10^−9^ M octopamine and 1 × 10^−4^ M IBMX. Relative change in fluorescence (corresponding to the amount of cAMP) is given as the percentage of the value obtained in the exclusive presence of 1 × 10^−9^ M octopamine (=100%). Data represent the mean ± SD of eight values from a typical experiment. All determinations were independently repeated at least three times.

**Figure 8:**
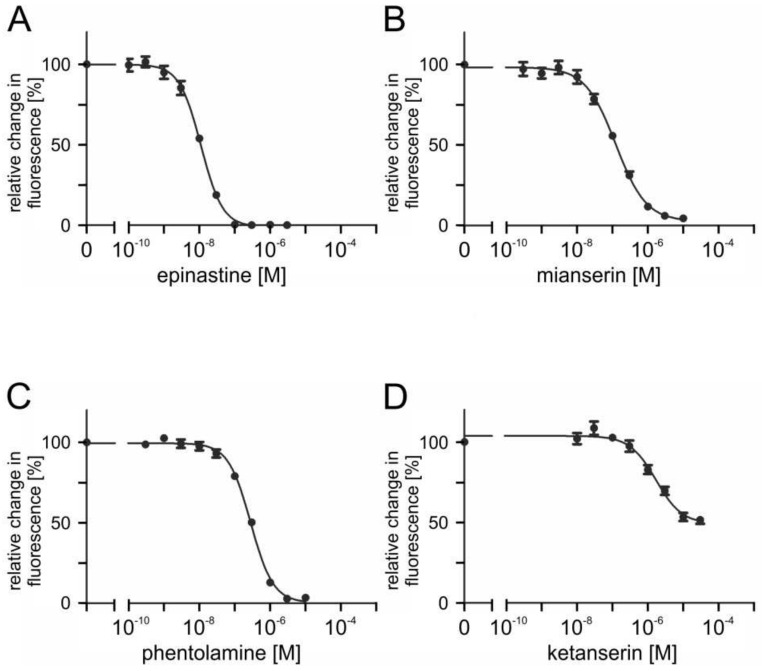
Effects of putative antagonists on tyramine-activated PaOctβ2R-HA. Concentration series of (**A**) epinastine, (**B**) mianserin, (**C**) phentolamine, and (**D**) ketanserin were applied in the presence of 1 × 10^7^ M tyramine and 1 × 10^−4^ M IBMX. Relative change in fluorescence (corresponding to the amount of cAMP) is given as the percentage of the value obtained in the exclusive presence of 1 × 10^−7^ M tyramine (=100%). Data represent the mean ± SD of eight values from a typical experiment. All determinations were independently repeated at least three times.

To assess the ability of potential antagonists to impair the activity of PaOctβ2R-HA, measurements with increasing concentrations of epinastine, mianserin, phentolamine, ketanserin, yohimbine, 5-carboxamidotryptamine (5-CT), 5-methoxytryptamine (5-MT), and 8-hydroxy-2-(dipropylamino)tetralin (8-OH-DPAT) were performed on non-saturating octopamine (1 × 10^−9^ M) and tyramine (1 × 10^−7^ M) concentrations, respectively. The reduction of cellular cAMP production, monitored as a decrease in Fluo-4 fluorescence (see Section 4.9), was quantified and normalized to the value obtained without adding antagonists (=100%). Normalized data were used to construct inhibitor concentration-response curves. Representative data are shown in Figure 7 and Figure 8.

Ligand concentrations that led to half-maximal inhibition of PaOctβ2R-HA (IC_50_) were determined from the concentration-response curves. The calculated IC_50_ values for each antagonist displacing either octopamine (1 × 10^−9^ M) or tyramine (1 × 10^−7^ M) are summarized in Table 2 and Table 3, respectively. The most potent antagonist on octopamine- and tyramine-stimulated PaOctβ2R-HA was epinastine with IC_50_ values of ~1.2 × 10^−8^ M and ~1.3 × 10^−8^ M, respectively. Low IC_50_ values were also obtained for mianserin and phentolamine (see Table 2 and Table 3).

Ketanserin reduced octopamine-induced receptor activity by approximately 80% but at much higher concentrations (IC_50_ ~5.1 × 10^−6^ M). Inhibition of tyramine-activated PaOctβ2R-HA was even less efficacious (50%; IC_50_ ~1.7 × 10^−6^ M). Only at the highest ligand concentration, 5-CT inhibited PaOctβ2R-HA activation by approximately 40%. Yohimbine, 5-MT, and 8-OH-DPAT did not impair octopamine- or tyramine-stimulated PaOctβ2R-HA activity.

Based on these data the same rank order of antagonist potency was uncovered for octopamine- and tyramine-stimulated PaOctβ2R-HA. The receptor was efficiently blocked by epinastine, mianserin, and phentolamine whereas yohimbine, 5-MT, and 8-OH-DPAT were non effective at all.

## 3. Discussion

There is ongoing interest in understanding the physiological and behavioral roles of octopaminergic signaling in insects (for recent reviews, see: [2,3,4,41,42,43]). An important step in meeting this challenge is to determine the molecular and functional-pharmacological properties of octopamine receptor subtypes. Here we describe the functional characterization of PaOctβ2R, the second octopamine receptor subtype of the cockroach, *P. americana*, an established model insect in neurobiological, physiological, and toxicological studies (for reviews, see: [10,44,45,46]). The primary structure of PaOctβ2R phylogenetically clusters with protostomian β-adrenergic-like octopamine receptors. Activation of PaOctβ2R by the phenolamines octopamine and tyramine led to a substantial increase in cAMP synthesis. Further candidates for biogenic amine receptors, including two additional β-adrenergic octopamine receptors, were identified by screening the transcriptome data of various cockroach tissues.

### 3.1. Structural Properties of the PaOctβ2R Protein and Phylogenetic Classification

Applying several in-silico analyses confirmed that PaOctβ2R is a member of the class A (rhodopsin-like) GPCR family. This assessment is supported by the presence of cognate amino acid residues and motifs within the TM segments in PaOctβ2R, e.g., N_383_PLIY in TM7 or the D_163_RY motif at the C-terminal end of TM3.

Most class A (rhodopsin-like) GPCRs are activated by ligands docking to specific residues in the binding pocket of the receptor near the extracellular side. Functionally important amino acid residues present in β-adrenergic-like octopamine receptors are well conserved in the PaOctβ2R sequence. These are an aspartic acid residue (D_146_) in TM3 and two of four closely grouped serine residues found in TM5 (S_235, 239_) (see: Supplementary Figure S1). Octopamine appears to bind via its amine group and its hydroxyl group to the aspartic acid and one of the serine residues of the receptor, respectively [47]. In addition, phenylalanine and/or tryptophan residues in TM6 and TM7 (see: Supplementary Figure S1) might contribute to π–π interaction with delocalized electrons in octopamine and stabilize the receptor ligand interaction.

The coupling of GPCRs to specific G proteins is brought about by amino-acid residues in close vicinity to the plasma membrane of the 2nd and 3rd intracellular loops and of the cytoplasmic C-terminus of the receptor [48,49]. Various insect β-adrenergic-like octopamine receptors possess strikingly similar amino-acid sequences throughout their 2nd cytoplasmic loops and in the vicinity of TM5 and TM6 within their 3rd cytoplasmic loops, regions largely determining the specificity of receptor/G_s_-protein coupling [50].

Our phylogenetic analysis including all major insect biogenic amine GPCR families resulted in a well-resolved phylogram (Figure 2). Protostomian β-adrenergic-like octopamine receptors seem to be closely related to deuterostomian β-adrenergic receptors and D_1_-like dopamine receptors (Figure 2), emphasizing the idea of “ligand-hopping” during evolution of aminergic GPCRs [38]. When new receptors evolved by gene duplication, they eventually needed new ligands for activation. Because of structural constrains, the only way to obtain “new” aminergic ligands was to repurpose already existing biogenic amines from other systems. The frequent ligand exchanges during evolution of aminergic GPCRs strongly contrasts with the situation observed for neuropeptide and protein hormone GPCRs, where generally co-evolution between receptors and their ligands takes place [38,51,52].

### 3.2. Posttranslational Modification of PaOctβ2R

Posttranslational modifications in intracellular loops of PaOctβ2R, like phosphorylation, may affect the signaling properties of the protein. Cysteine residues in the C-terminus of different biogenic amine receptors were found to undergo posttranslational palmitoylation [53]. This modification generates a fourth intracellular loop that participates in receptor-G protein binding. PaOctβ2R has three such cysteine residues in its C-terminal end. In addition, the primary structure of PaOctβ2R harbors two potential sites for N-linked glycosylation (Figure S1) and we were able to show that at least the heterologously expressed receptor protein is present to a considerable extent in glycosylated form (Figure S2).

### 3.3. Pharmacological Properties of the PaOctβ2R Protein

The PaOctβ2R receptor was functionally expressed in flpTM cells. Coupling of PaOctβ2R to intracellular signaling cascades was examined via cell-endogenous G-proteins. PaOctβ2R, like other β-adrenergic-like octopamine receptors from insects [24,25,26,37,54,55] and mammalian β-adrenergic receptors (for a review, see: [56]), is positively coupled to adenylyl cyclase via G_s_ proteins, and causes an increase in [cAMP]_i_. With a mean EC_50_ of 4.67 × 10^−10^ M, activation of PaOctβ2R was much more sensitive to octopamine than to tyramine (mean EC_50_ = 4.30 × 10^−8^ M; Table 1). These data agree well with those described for orthologous receptors [25,26,37].

Inhibition of PaOctβ2R-mediated increase in [cAMP]_i_ in the cell line was examined with various synthetic antagonists. In addition to epinastine (IC_50_ = 1.2 × 10^−8^ M/1.3 × 10^−8^ M), which is a histamine H_1_ receptor antagonist in vertebrates but also an antagonist of insect octopamine receptors [57,58], the action of octopamine/tyramine on PaOctβ2R could be blocked by the non-selective α-adrenergic antagonist phentolamine (IC_50_ = 1.0 × 10^−7^ M/1.16 × 10^−7^ M). In addition, classical serotonergic ligands, e.g., the non-selective 5-HT_2_ receptor antagonist mianserin (IC_50_ = 2.57 × 10^−7^ M/2.56 × 10^−7^ M) and the selective 5-HT_2A_ antagonist ketanserin (IC_50_ = 5.12 × 10^−6^ M/1.68 × 10^−6^ M), were also potent blockers of the action of octopamine or tyramine on PaOctβ2R. In particular mianserin is known to also act as a potent antagonist at insect octopamine receptors [23,59,60] and was found to be an antagonist of certain insect tyramine receptor of the honeybee as well [18,30].

### 3.4. Octopamine Receptors as Molecular Targets of Insecticides

As mentioned above, octopamine signaling in insects is highly complex. Octopamine receptors characterized so far can be classified as either α_1_-adrenergic-like which preferentially couple to G_q_ proteins and induce intracellular Ca^2+^ mobilization, α_2_-adrenergic-like which preferentially couple to G_i_ proteins and inhibit adenylyl cyclase activity, or β-adrenergic-like which preferentially couple to G_s_ proteins and activate adenylyl cyclases [2,3,19,22,23]. Octopamine receptors, especially those that lead to an increase in [cAMP]_i_, are also potential molecular targets of selectively acting insecticides, e.g., from the class of formamides and plant essential oils (for reviews, see: [61,62,63]). As early as in the middle of the 1980s, the formamidines demethylchlordimeform (DMCD), BTS-27271, and amitraz were shown to mimic the action of octopamine in elevating adenylyl cyclase activity in the nervous tissue of *P. americana* [64,65]. In *D. melanogaster*, however, DMCD and chlordimeform inhibit octopamine-stimulated adenylyl cyclase activity, whereas amitraz activates the enzyme [66]. Dihydrooxadiazine insecticides were reported to elevate cAMP levels in homogenates of the *P. americana* nervous system most likely by stimulation of octopamine receptors [67]. The neurotoxic activity of plant essential oils and their purified constituents against various insect species is also likely due to their interactions with octopamine and/or tyramine receptors [68,69,70]. Recently, Kita and colleagues [71] systematically performed pharmacological studies using heterologously expressed octopamine receptors of the silkworm *Bombyx mori* to identify the molecular target of the formamidine amitraz. The β-adrenergic-like octopamine receptor BmOAR2 [37] turned out to be more sensitive to amitraz and its metabolite DPMF than the α1-adrenergic-like octopamine receptor BmOA2 [71,72]. Moreover, DPMF was confirmed to be a more potent octopamine receptor agonist than amitraz itself, indicating that amitraz undergoes metabolic activation. Site-directed mutagenesis studies clearly showed that amitraz and DPMF act as orthosteric antagonists [73]. These groundbreaking studies provided evidence that target-site insensitivity significantly contributes to the widespread amitraz resistance [73]. In veterinary medicine, amitraz is also used to control ectoparasitic mites, e.g., the cattle tick *Rhipicephalus microplus* [74,75] and *Varroa destructor*, a mite that attacks and feeds on honeybees [76,77]. A non-synonymous SNP in a β-adrenergic-like octopamine receptor of amitraz-resistant *R. microplus* was proposed to be the cause of amitraz resistance [78]. In an elegant study, Guo and colleagues [79] found that amitraz and DPMF activated various *V. destructor* octopamine receptors. The mite VdOctβ2R was more sensitive to amitraz and its metabolite than the honeybee AmOctβ2R [79]. Furthermore, replacement of three bee-specific residues with their counterparts in the mite receptor increased amitraz sensitivity of the bee AmOctβ2R receptor, suggesting that these three residues are responsible for the resistance of honeybees to amitraz [79]. Last but not least, behavioral studies using *D. melanogaster* null mutants of octopamine receptors identified the DmOctβ2R as the sole target of amitraz in the fly [79].

The American cockroach *P. americana* is considered to be an insect pest of significant public health importance. They can passively transport pathogenic bacteria, such as *Salmonella*, on their body surfaces, particularly in areas like hospitals [80]. In addition, house dust containing cockroach feces and body parts can trigger allergic reactions and asthma in certain individuals [81,82]. For these reasons, cockroach populations are controlled using insecticides [83]. With the characterization of the PaOctβ2R described here and in particular the production of a cell line that stably expresses this receptor, we now have an efficient tool to screen for potential and highly selective insecticides. Further insecticide targets can possibly be found among the receptors that we have identified in the transcriptomes.

## 4. Materials and Methods

### 4.1. Amplification of a Cockroach β-Adrenergic-Like Octopamine Receptor (PaOctβ2R) cDNA

Based on sequence conservation throughout various arthropod species, degenerate primers (DF1: 5′-TGYTGGBTICCITTYTT-3′; DR1: 5′-TTDATISHRTADATIAYIGGRTT-3′) corresponding to highly conserved amino acid sequences in TM 6 [CW(L/V)PFF] and TM 7 [NP(V/I)IY(T/A/S)IF] of biogenic amine receptors were designed to amplify receptor fragments [10,27,28,29,30]. Polymerase chain reaction (PCR) was performed on a *P. americana*-brain cDNA library [84] under the following conditions: 1 cycle of 2.5 min at 94 °C, followed by 35 cycles of 40 s at 94 °C, 40 s at 45 °C, and 20 s at 72 °C, and a final extension of 10 min at 72 °C. The PCR products were cloned into pGEM-T vector (Promega, Mannheim, Germany) and subsequently analyzed by DNA sequencing (AGOWA, Berlin, Germany). Missing 5′- and 3′-regions of the cDNA were amplified by SMART RACE (rapid amplification of cDNA ends) experiments (Clontech, Saint-Germain-en-Laye, France). Finally, a PCR was performed on single-stranded *P. americana*-brain cDNA to amplify the entire coding region of PaOctβ2R by using gene-specific primers annealing in the 5′- and 3′-untranslated regions (SF: 5′-CGTCGTGAACCTCTGACATC-3′; SR: 5′-CCTCAAGTCGGGTAACTGTC-3′). The nucleotide sequence of PaOctβ2R has been submitted to the GenBank database (accession#: OL739164). N-glycosylation sites were predicted by NetNGlyc 1.0 Server (http://www.cbs.dtu.dk/services/NetNGlyc/ (accessed on 10 September 2021)). Putative phosphorylation sites were predicted by NetPhos 3.1 Server (http://www.cbs.dtu.dk/services/NetPhos/ (accessed on 10 September 2021); [85]).

### 4.2. Transcriptome Sequencing and De novo Assembly of Nucleotide Sequences

Various tissues from adult *P. americana* (CNS, frontal ganglion, corpora cardiaca, heart, leg muscle, midgut) were prepared as described in [86]. Subsequent RNA isolation, cDNA preparation and Illumina Next Generation Sequencing were carried out by BGI (Beijing Genomics Institute, New Territories, Hong Kong, China). Each library was sequenced for 100 bp paired ends, the resulting RAW reads were filtered by removing adapter sequences, contamination, and low-quality reads. De novo assembly of RNA-Seq data was conducted by using Trinity (v2.2.0) [87]. The local BLAST tool of BioEdit v. 7.0.5.3 [40] was used to search for orthologous sequences of cockroach monoaminergic receptors, with receptor sequences of *A. mellifera* and *D. melanogaster* as queries [38].

### 4.3. Multiple Sequence Alignment and Phylogenetic Analysis

For phylogenetic analysis, we included amino acid sequences of biogenic amine receptors of various protostomian and deuterostomian species. Sequences were obtained from NCBI databases (NCBI, Bethesda, MD, USA). Multiple sequence alignments were performed with BioEdit and amino acid sequences were trimmed to regions encoding TM 1-4, TM 5, TM 6, and TM 7. Afterwards, evolutionary analyses were conducted in MEGA7 [88]. The evolutionary history was inferred by using the Maximum Likelihood method based on the Poisson correction model. Initial tree(s) for the heuristic search were obtained automatically by applying Neighbor-Join and BioNJ algorithms to a matrix of pairwise distances estimated using a JTT model, and then selecting the topology with superior log likelihood value. The analysis involved 93 amino acid sequences. There was a total of 238 positions in the final dataset. The human rhodopsin sequence (HsRHOD) formed the outgroup.

Sequence identity and similarity of β_2_-adrenergic-like octopamine receptors between *P. americana*, *Cryptotermes secundus*, *D. melanogaster*, and *A. mellifera* were determined by using the BLOSUM62 similarity matrix.

### 4.4. RT-PCR Amplification of PaOctβ2R Fragments

RT-PCR experiments to determine the tissue distribution of PaOctβ2R were performed as described earlier [27,28]. Briefly, receptor-specific fragments were amplified from 100 ng total RNA isolated from brain, salivary glands, midgut, Malpighian tubules, and leg muscle of adult male cockroaches. Sense and antisense primers were 5′-CTCAACCGCTTCCATCCTCC-3′ (RT-F) and 5′-GCTTCTCTTGCCTGTTCGCC-3′ (RT-R), respectively. Amplification resulted in fragments of the expected length of 344 bp. cDNA was synthesized for 30 min at 50 °C followed by a denaturation step at 94 °C for 2 min. Amplification was performed for 35 cycles at 94 °C for 40 s, 66 °C for 40 s, and 72 °C for 30 s, followed by a final extension at 72 °C for 10 min. For reamplification, 2 μL of the PCR product of the initial RT-PCR were used as a template. RT-PCR was also performed with actin-specific primers (Accession No. AY116670) as an internal control (ActinF: 5′-CGAGTAGCTCCTGAAGAGC-3′; ActinR: 5′-GGCCTCTGGACAACGGAACC-3′; fragment length: 488 bp).

### 4.5. Construction of PaOctβ2R-HA Expression Vector

An expression-ready construct of *PaOctβ2R* in pcDNA3.1(+) vector was generated by PCR. Specifically, receptor encoding cDNA was modified in a PCR with primers PaOctβ2R-expr-F (5′-TTTAAGCTTCCACCATGGCGTCCAATCCCGATATC-3′) and PaOctβ2R-expr-R (5′-TTTGAATTCCAGACTGCTGCCGAACTCGC-3′). In front of the start codon, a HindIII restriction site (AAGCTT) and a Kozak consensus motif (CCACC; [89]) were inserted. The stop codon was replaced by an EcoRI recognition sequence (GAATTC). We reused the pc*Am5-ht1A*-HA construct [90] and exchanged the *Am5-ht1A* cDNA for the Pa*octβ2R* cDNA by ligation into the HindIII and EcoRI sites. The resulting construct (pc*Paoctβ2R*-HA) is extended in frame at the 3′ end with a sequence encoding the hemagglutinin A (HA) tag (YPYDVPDYA) which allowed us to monitor receptor protein expression using a specific anti-HA antibody (Roche Applied Science/Sigma-Aldrich/Merck, Mannheim, Germany). The correct insertion was confirmed by DNA sequencing.

### 4.6. Functional Expression of the PaOctβ2R-HA Receptor

For PaOctβ2R-HA expression and functional analysis, we used a human embryonic kidney (HEK293; flpIn cells; Invitrogen/ThermoFisher Scientific; #750-07)-based cell line that had been transfected with a gene encoding a variant of the A2-subunit of the olfactory cyclic nucleotide-gated ion channel (CNG; [91]; flpTM cells, provided by Sibion biosciences, Jülich, Germany). Cells were transfected with 10 µg of the pc*PaOctβ2R*-HA construct by a modified calcium phosphate method [92] following a previously established protocol [93]. Transfected cells were selected in the presence of the antibiotics G418 (1 mg/mL) and hygromycin (100 µg/mL).

### 4.7. Immunofluorescent Staining of Cell Lines

Cells were grown on cover slips in 24 well plates and fixed with 4% (*v/v*) paraformaldehyde for 10 min at room temperature (RT). After several rinses with PBS, samples were blocked for 1 h at RT in blocking solution (CT: 0.5% (*v/v*) Triton X-100, 5% (*v/v*) ChemiBLOCKER (Merck, Darmstadt, Germany)). Subsequently, samples were incubated with primary antibodies (rat anti-HA (Roche/Sigma-Aldrich/Merck) dilution 1:100; mouse anti-CNG [23] dilution 1:200) in CT at 4 °C over night, rinsed for several times with PBS and then incubated with secondary antibodies (goat anti-rat-Alexa488 (1:500), Invitrogen/Thermo Fisher Scientific, Dreieich, Germany (#A11006); donkey anti-mouse-Cy3 (1:400), Dianova, Hamburg, Germany (715-165-150)) in CT at RT for 1 h. Nuclei were stained with TOPRO-3 (dilution 1:1000; Invitrogen/Thermo Fisher Scientific). Finally, samples were washed with PBS, before mounting the coverslips containing cells in Aqua-Poly/Mount (Polysciences, Eppelheim, Germany) on microscope slides. Fluorescent images were obtained with an inverted confocal microscope (TCS SP5II; Leica, Wetzlar, Germany).

### 4.8. Western Blot Analysis

Membrane proteins from PaOctβ2R-HA expressing cells and non-transfected flpTM cells were prepared as described previously [23,26]. Cells were lysed in 10 mM NaCl, 25 mM HEPES pH 7.5, 2 mM EDTA and a mammalian protease inhibitor cocktail diluted 1:500 (mPIC; Sigma-Aldrich/Merck). After centrifugation, membrane proteins were solubilized in 100 mM NaCl, 25 mM HEPES pH 7.5, mPIC (1:500 dilution) and 1% (*w/v*) (3-((3-cholamidopropyl)-dimethylammonio)-1-propanesulfonate, (CHAPS)). Proteins (30 µg per lane) were separated by sodium dodecyl sulfate polyacrylamide gel electrophoresis (SDS-PAGE; 10% gel) and transferred onto polyvinylidene fluoride membrane (PVDF, Merck/Millipore, Darmstadt, Germany). Non-specific binding sites were blocked by incubation for 45 min in 5% (*w/v*) dry milk in phosphate buffered saline (PBS; 130 mM NaCl, 7 mM Na_2_HPO_4_, 3 mM NaH_2_PO_4_, pH 7.4) at room temperature. The membrane was incubated with primary antibodies (anti-HA, dilution 1:1000; Roche/Sigma-Aldrich/Merck) in PBS containing 0.02% (*v/v*) Tween-20 (PBT) overnight at 4 °C. After rinsing the membrane three times with PBT for 15 min each, secondary antibodies conjugated to horseradish peroxidase (donkey anti-rat-HRP, dilution 1:5000 (Sigma-Aldrich/Merck) in PBT containing 0.5% (*w/v*) dry milk were added for 1 h at room temperature. After rinsing the membrane three times with PBT for 15 min each, and two times with PBS for 15 min each, signals were visualized with an enhanced chemiluminescence detection system (Western Bright™-Kit; Advansta; San Jose, CA, USA) on Hyperfilm™ ECL (GE Healthcare/Merck, Darmstadt, Germany). Staining of the Western blot against the CNG channel followed the same protocol, except that a monoclonal primary (mouse anti-CNG; dilution 1:500; [23]) and rabbit anti-mouse secondary antibody (dilution 1:80,000, Sigma-Aldrich/Merck) was used.

### 4.9. Functional Analysis of the PaOctβ2R-HA Receptor

A stably transfected cell line was established to examine PaOctβ2R-HA receptor activity by Ca^2+^ imaging. Control measurements were performed in the parental (flpTM) cell line. Changes in [cAMP]_i_ were registered indirectly via co-expressed CNG channels that are opened by cAMP and cause an influx of extracellular Ca^2+^ [18,26,91]. Changes in [Ca^2+^]_i_ were monitored with the Ca^2+^-sensitive fluorescent dye Fluo-4. Cells were grown in 96-well dishes to a density of approximately 2 × 10^4^ cells per well and were loaded at room temperature with Fluo-4 AM as described previously [26]. After 90 min, the loading solution was substituted for dye-free extracellular solution (ECS; 120 mM NaCl, 5 mM KCl, 2 mM MgCl_2_, 2 mM CaCl_2_, 10 mM HEPES, and 10 mM glucose, pH 7.4 (NaOH)) containing 100 µM 3-isobutyl-1-methylxanthine (IBMX; Sigma-Aldrich/Merck, Darmstadt, Germany) as an inhibitor of cell-endogenous phosphodiesterases. The plate was transferred into a fluorescence reader (FLUOstar Omega, BMG Labtech, Ortenberg, Germany) to monitor Fluo-4 fluorescence. The excitation wavelength was 485 nm. Fluorescence emission was detected at 520 nm. Concentration series of various biogenic amines and synthetic receptor ligands were added once Fluo-4 fluorescence had reached a stable value in each well. The changes in Fluo-4 fluorescence were recorded automatically. Concentration-response curves were established from at least three independent experiments. Data points were derived from four-fold or eightfold determination. Data were analyzed and displayed using Prism 5.04 software (GraphPad, San Diego, CA, USA).

## Figures and Tables

**Table 1:** Mean values for half-maximal stimulation (EC_50_ [M] and pEC_50_ ± SD) for octopamine and tyramine on PaOctβ2R-HA. Values were obtained from non-linear fitting of the data (n = number of experiments) from concentration-response curves (GraphPad Prism 5.04).

	Octopamine (n = 5)	Tyramine (n = 5)
EC_50_ [M]	4.67 × 10^−10^	4.30 × 10^−8^
pEC_50_	9.35 ± 0.04	7.39 ± 0.04

**Table 2:** Mean values for half-maximal inhibition (IC_50_ [M] and pIC_50_ ± SD) for substances with antagonistic activity on octopamine-activated PaOctβ2R-HA. For each substance, at least three independent experiments (n) with octuplicate measurements were performed. Values were obtained from non-linear fitting of the data from concentration-response curves (GraphPad Prism 5.04). The maximal inhibition of PaOctβ2R-HA activity is given in [%].

Substance	IC_50_ [M]	pIC_50_	n	Maximal Inhibition [%]
epinastine	1.2 × 10^−8^	7.944 ± 0.042	4	100
mianserin	1.0 × 10^−7^	7.006 ± 0.055	3	100
phentolamine	2.57 × 10^−7^	6.608 ± 0.069	4	100
ketanserin	5.12 × 10^−6^	5.291 ± 0.048	3	80
5-CT	n.d.	n.d.	6	40
yohimbine	-	-	3	-
5-MT	-	-	3	-
8-OH-DPAT	-	-	3	-

**Table 3:** Mean values for half-maximal inhibition (IC_50_ [M] and pIC_50_ ± SD) for substances with antagonistic activity on tyramine activated PaOctβ2R. For each substance, at least three independent experiments (n) with octuplicate measurements were performed. Values were obtained from non-linear fitting of the data from concentration-response curves (GraphPad Prism 5.04). The maximal inhibition of PaOctβ2R-HA activity is given in [%].

Substance	IC_50_ [M]	pIC_50_	n	Maximal Inhibition [%]
epinastine	1.3 × 10^−8^	7.951 ± 0.042	4	100
mianserin	1.16 × 10^−7^	6.938 ± 0.058	3	100
phentolamine	2.56 × 10^−7^	6.594 ± 0.022	4	100
ketanserin	1.68 × 10^−6^	5.766 ± 0.097	3	50
5-CT	n.d.	n.d.	6	40
yohimbine	-	-	3	-
5-MT	-	-	3	-
8-OH-DPAT	-	-	3	-

## Supplementary Materials

The following supporting information can be downloaded at https://www.mdpi.com/article/10.3390/ijms23031677/s1.
