# Supplementary material for: PaOctβ2R: Identification and Functional Characterization of an Octopamine Receptor Activating Adenylyl Cyclase Activity in the American Cockroach Periplaneta americana
Source: Int J Mol Sci. 2022 Jan 31;23(3):1677. doi: 10.3390/ijms23031677 (PMC8835733; doi:10.3390/ijms23031677)
Supplement: Supplementary file 1 [file ijms-23-01677-s001.zip › ijms-1568167-supplementary.pdf]

Article

PaOctβ2R: identification and functional characterization of an octopamine receptor activating adenylyl cyclase activity in the American cockroach *Periplaneta americana*

Wolfgang Blenau, Anna-Sophie Bremer, Yannik Schwietz, Daniel Friedrich, Lapo Ragionieri, Reinhard Predel, Sabine Balfanz and Arnd Baumann

Supplementary Material

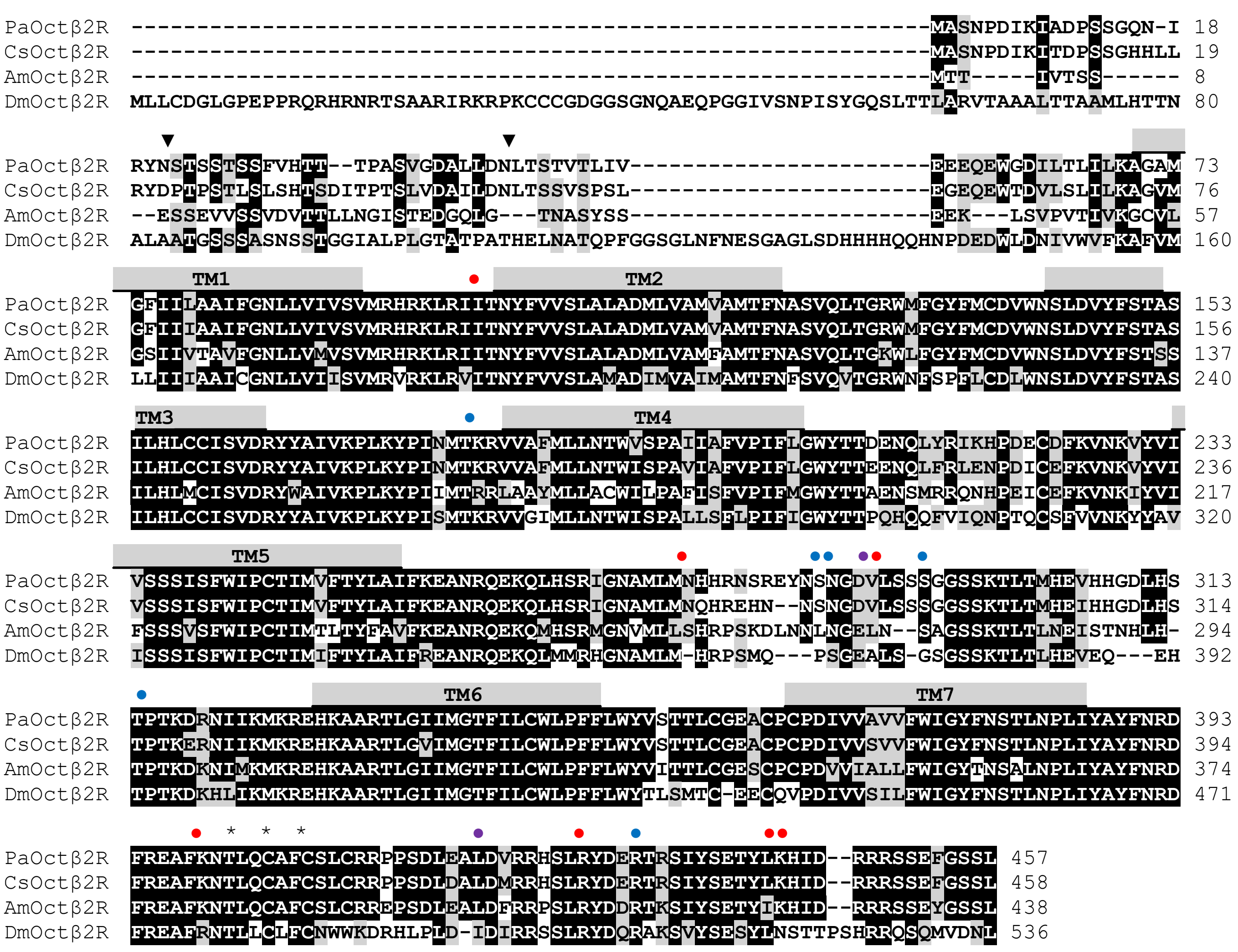

Figure S1. Amino acid sequence alignment of PaOctβ2R and β2-adrenergic-like octopamine receptors from the drywood termite *Cryptotermes secundus* (CsOctβ2R; XP_023721065.1), the honey bee *Apis mellifera* (AmOctβ2R; XP_006558130.1), and the vinegar fly *Drosophila melanogaster* (DmOctβ2R; CG33976; NP_001034049.1). Identical residues (≥75%) are shown as white letters against black, whereas conservatively substituted residues are shaded. Grey bars indicate putative transmembrane domains (TM1-7). Potential posttranslational modification sites are labeled: N-glycosylation (▼), protein kinase A (PKA) phosphorylation (●), protein kinase C (PKC) phosphorylation (●), phosphorylation by both PKA and PKC (●). N-glycosylation sites were predicted by NetNGlyc 1.0 Server (http://www.cbs.dtu.dk/services/NetNGlyc/). Putative phosphorylation sites were predicted by NetPhos 3.1 Server (http://www.cbs.dtu.dk/services/NetPhos/). The amino acid position is given on the right.

Table S1. Accession numbers and annotations of sequences used in the phylogenetic analyses.

ID	Accession No	Species	Description	Reference
**PROTOSTOMIA**, **Lophotrochozoa**, Annelida				
Pdα1	APC23842.1	*P. dumerilii*	α_1-adrenergic receptor	[1]
Pdα2	APC23843.1	*P. dumerilii*	α_2-adrenergic receptor	[1]
PdTAR1	AKQ63052.1	*P. dumerilii*	tyramine receptor 1	[1]
PdTAR2	APC23184.1	*P. dumerilii*	tyramine receptor 2	[1]
PdOctα1R	APC23183.1	*P. dumerilii*	α_1-adrenergic-like octopamine receptor	[1]
PdOctβR	APC23841.1	*P. dumerilii*	β-adrenergic-like octopamine receptor	[1]
**PROTOSTOMIA**, **Ecdysozoa**, Priapulida				
Pcα1	XP_014662992.1	*P. caudatus*	α_{1A}-adrenergic receptor-like	
Pcα2	XP_014681069.1	*P. caudatus*	α_{2C}-adrenergic receptor-like	
**PROTOSTOMIA**, **Ecdysozoa**, Arthropoda				
PaTAR1A	CAQ48240.1	*P. americana*	tyramine receptor 1A	[2]
PaTAR1B	SNT95699.1	*P. americana*	tyramine receptor 1B	[3]
PaTAR2	OL739162	*P. americana*	tyramine receptor 2	This work
PaOctα1R	AAP93817.1	*P. americana*	α_1-adrenergic-like octopamine receptor	[4]
PaOctα2R	OL904962	*P. americana*	α_2-adrenergic-like octopamine receptor	This work
PaOctβ1R fragment	OL739163	*P. americana*	β-adrenergic-like octopamine receptor	This work
PaOctβ2R	OL739164	*P. americana*	β-adrenergic-like octopamine receptor	This work
PaOctβ3R fragment	OL739165	*P. americana*	β-adrenergic-like octopamine receptor	This work
PaDOP1	OL770093	*P. americana*	D_1-like dopamine receptor	This work
PaDOP2A	CDK37789.1	*P. americana*	invertebrate-type dopamine receptor, isoform A	[5]
PaDOP3	OL739166	*P. americana*	D_2-like dopamine receptor	This work
PaDOPEcR	OL739167	*P. americana*	dopamine/ecdysteroid receptor	This work
Pa5-HT1	CAX65666.1	*P. americana*	5-HT_1-like serotonin receptor	[6]
Pa5-HT2α	OL739168	*P. americana*	5-HT_2-like serotonin receptor	This work
Pa5-HT2β	OL739169	*P. americana*	5-HT_2-like serotonin receptor	This work
Pa5-HT7	OL739170	*P. americana*	5-HT_7-like serotonin receptor	This work
Pa18	OL739171	*P. americana*	orphan	This work
PaBAR X	OL739172	*P. americana*	orphan	This work
AmTAR1	NP_001011594.1	*A. mellifera*	tyramine receptor 1	[7]
AmTAR2	APL96716.1	*A. mellifera*	tyramine receptor 2	[8]
AmOctα1R	NP_001011565.1	*A. mellifera*	α_1-adrenergic-like octopamine receptor	[9]
AmOctα2R	XP_001122075.3	*A. mellifera*	α_2-adrenergic-like octopamine receptor	[10]
AmOctβ1R	CCO13922.1	*A. mellifera*	β-adrenergic-like octopamine receptor 1	[11]
AmOctβ2R	CCO13923.1	*A. mellifera*	β-adrenergic-like octopamine	[11]

			receptor 2	
AmOctβ3R	CCO13924.1	*A. mellifera*	β-adrenergic-like octopamine receptor 3	[11]
AmOctβ4R	CCO13925.1	*A. mellifera*	β-adrenergic-like octopamine receptor 4	[11]
AmDOP1	NP_001011595.1	*A. mellifera*	D_1-like dopamine receptor	[12]
AmDOP2	NP_001011567.1	*A. mellifera*	invertebrate-type dopamine receptor	[13, 14]
AmDOP3	NP_001014983.1	*A. mellifera*	D_2-like dopamine receptor	[15]
AmDopEcR	XP_016768310.1	*A. mellifera*	dopamine/ecdysteroid receptor	
Am5-HT1A	CBI75449.1	*A. mellifera*	5-HT_1-like serotonin receptor	[16]
Am5-HT2α	CBX90120.1	*A. mellifera*	5-HT_2-like serotonin receptor	[17]
Am5-HT2β	CBX90121.1	*A. mellifera*	5-HT_2-like serotonin receptor	[17]
Am5-HT7	CAJ28210.1	*A. mellifera*	5-HT_7-like serotonin receptor	[18]
Am18	XP_026297873	*A. mellifera*	orphan	
DmTAR1	NP_524419.2	*D. melanogaster*	tyramine receptor 1 (octopamine-tyramine receptor)	[19]
DmTAR2	NP_650652.1	*D. melanogaster*	tyramine receptor 2	[20]
DmTAR3	NP_650651.1	*D melanogaster*	tyramine receptor 3	[20, 21]
DmOctα1AR	CAB38026.1	*D. melanogaster*	α_1-adrenergic-like octopamine receptor 1, splice variant 1A	[22]
DmOctα1BR	CAB38025.1	*D. melanogaster*	α_1-adrenergic-like octopamine receptor 1, splice variant 1B	[22]
DmOctα2R	NP_650754.2	*D. melanogaster*	α_2-adrenergic-like octopamine receptor	[23]
DmOctβ1R	NP_651057.1	*D. melanogaster*	β-adrenergic-like octopamine receptor 1	[22, 24]
DmOctβ2R	NP_001034049.1	*D. melanogaster*	β-adrenergic-like octopamine receptor 2	[24]
DmOctβ3R	NP_001034043.2	*D. melanogaster*	β-adrenergic-like octopamine receptor 2	[24]
DmDOP1	CAA54451.1	*D. melanogaster*	D_1-like dopamine receptor	[25]
DmDOP2	NP_733299.1	*D. melanogaster*	invertebrate-type dopamine receptor	[26]
DmDOP3	AAX52464.2	*D. melanogaster*	D_2-like dopamine receptor	[27]
DmDOPEcR	NP_001014559.1	*D. melanogaster*	dopamine/ecdysteroid receptor	[28]
Dm5-HT1A	CAA77570.1	*D. melanogaster*	5-HT_1-like serotonin receptor	[29]
Dm5-HT1B	CAA77571.1	*D. melanogaster*	5-HT_1-like serotonin receptor	[29]
Dm5-HT2α	NP_524223.2	*D. melanogaster*	5-HT_2-like serotonin receptor	[30]
Dm5-HT2β	NP_001262373.1	*D. melanogaster*	5-HT_2-like serotonin receptor	[31]
Dm5-HT7	NP_524599.1	*D. melanogaster*	5-HT_7-like serotonin receptor	[32]
DmCG13579	NP_611917.2	*D. melanogaster*	orphan	
**DEUTEROSTOMIA**, **Ambulacraria**, Priapulida				
Skα1	ALR88680.1	*S. kowalevskii*	α_1-adrenergic receptor-like 067	[33]
Skα2	XP_002734932.1	*S. kowalevskii*	α_{2C}-adrenergic receptor-like	
SkTAR1	XP_002742354.2	*S. kowalevskii*	tyramine receptor 1	
SkTAR2A	XP_002734062.1	*S. kowalevskii*	tyramine receptor 2A	
SkTAR2B	XP_006812999.1	*S. kowalevskii*	tyramine receptor 2B	
SkOctα1R	XP_006823182.1	*S. kowalevskii*	α_1-adrenergic-like octopamine receptor	
SkOctβR	XP_002733926.1	*S. kowalevskii*	β-adrenergic-like octopamine receptor	

DEUTEROSTOMIA, **Chordata**, Vertebrata

Hsα1A	NP_000671.2	*H. sapiens*	α_{1A}-adrenergic receptor	[34]
Hsα1B	NP_000670.1	*H. sapiens*	α_{1B}-adrenergic receptor	[35]
Hsα1D	NP_000669.1	*H. sapiens*	α_{1D}-adrenergic receptor	[36]
Hsα2A	NP_000672.3	*H. sapiens*	α_{2A}-adrenergic receptor	[37]
Hsα2B	NP_000673.2	*H. sapiens*	α_{2B}-adrenergic receptor	[38]
Hsα2C	NP_000674.2	*H. sapiens*	α_{2C}-adrenergic receptor	[39]
Hsβ1	NP_000675.1	*H. sapiens*	β_1-adrenergic receptor	[40]
Hsβ2	NP_000015.1	*H. sapiens*	β_2-adrenergic receptor	[41]
Hsβ3	NP_000016.1	*H. sapiens*	β_3-adrenergic receptor	[42]
HsD1A	NP_000785.1	*H. sapiens*	D_{1A} dopamine receptor	[43]
HsD1B	NP_000789.1	*H. sapiens*	D_{1B} dopamine receptor	[44]
HsD2	NP_000786.1	*H. sapiens*	D_2 dopamine receptor isoform long	[45]
HsD3	NP_000787.2	*H. sapiens*	D_3 dopamine receptor isoform a	[46]
HsD4	NP_000788.2	*H. sapiens*	D_4 dopamine receptor	[47]
Hs5-HT1A	NP_000515.2	*H. sapiens*	5-HT_{1A} serotonin receptor	[48]
Hs5-HT1B	NP_000854.1	*H. sapiens*	5-HT_{1B} serotonin receptor	[49]
Hs5-HT1D	NP_000855.1	*H. sapiens*	5-HT_{1D} serotonin receptor	[50]
Hs5-HT1E	NP_000856.1	*H. sapiens*	5-HT_{1E} serotonin receptor	[51]
Hs5-HT1F	NP_000857.1	*H. sapiens*	5-HT_{1F} serotonin receptor	[52]
Hs5-HT2A	NP_000612.1	*H. sapiens*	5-HT_{2A} serotonin receptor isoform 1	[53]
Hs5-HT2B	NP_000858.3	*H. sapiens*	5-HT_{2B} serotonin receptor isoform 1	[54]
Hs5-HT2C	NP_000859.1	*H. sapiens*	5-HT_{2C} serotonin receptor 2C isoform a precursor	[53]
Hs5-HT7	NP_000863.1	*H. sapiens*	5-HT_7 serotonin receptor isoform a	[55]
HsRHOD	AAC31763.1	*H. sapiens*	rhodopsin	[56]

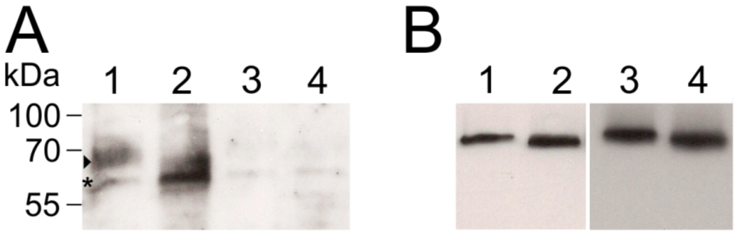

Figure S2. Expression of PaOctβ2R-HA in flpTM cells. A) Western blot of membrane proteins (30 μg) from flpTM + PaOctβ2R-HA expressing (lane 1, 2) and flpTM cells (lane 3, 4) were not treated (lane 1, 3) or treated with PNGase F (lane 2, 4). Proteins were separated by sodium dodecyl sulfate-polyacrylamide gel electrophoresis (SDS-PAGE) and blotted to a polyvinylidene difluoride (PVDF) membrane. The blot was probed with a rat anti-HA antibody (dilution 1:1,000). A secondary HRP-coupled donkey-anti-rat antibody (dilution 1:5,000) was used for detection. Bands were visualized with ECL. **B)** The same blot was subsequently probed with an antibody directed against the C-terminus of the CNG channel (dilution 1:500). A secondary HRP-coupled rabbit-anti-mouse antibody (dilution 1:80,000) was used for detection. Note that exposure times in (**B**) were 1 min (lanes 1, 2) and 2 s (lanes 3, 4). The sizes of marker proteins in kDa are given on the left margin.

Table S2. Detection of biogenic amine receptor sequences in transcriptomes of various cockroach tissues.

Receptor	CNS	Frontal ganglion	Corpora cardiaca	Heart	Leg muscle	Midgut
PaTAR1A	+	+	+	+	+	-
PaTAR1B	-	+	+	-	-	-
PaTAR2	+	+	+	+	-	+
PaOctα1R	+	+	+	+	+	+
PaOctα2R	-	+	+	-	-	-
PaOctβ1R	-	+	+	-	-	-
PaOctβ2R	+	+	+	+	+	-
PaOctβ3R	-	+	+	-	-	-
PaDOP1	-	+	+	-	-	-
PaDOP2	+	-	-	-	-	-
PaDOP3	-	+	+	-	-	-
PaDOPEcR	+	+	+	+	-	-
Pa5-HT1	+	+	+	-	-	+
Pa5-HT2α	+	+	+	+	-	+
Pa5-HT2β	+	+	+	+	-	-
Pa5-HT7	+	+	+	+	-	+
Pa18	+	+	+	-	-	-
PaBAR X	+	+	+	+	-	-

References

1. Bauknecht, P.; Jékely, G. Ancient coexistence of norepinephrine, tyramine, and octopamine signaling in bilaterians. *BMC Biol.* **2017**, *15*, 6.
2. Rotte, C.; Krach, C.; Balfanz, S.; Baumann, A.; Walz, B.; Blenau, W. Molecular characterization and localization of the first tyramine receptor of the American cockroach (*Periplaneta americana*). *Neuroscience* **2009**, *162*, 1120-1133.
3. Blenau, W.; Balfanz, S.; Baumann, A. PeaTAR1B: Characterization of a second type 1 tyramine receptor of the American cockroach, *Periplaneta americana*. *Int. J. Mol. Sci.* **2017**, *18*, 2279.
4. Bischof, L.J.; Enan, E.E. Cloning, expression and functional analysis of an octopamine receptor from *Periplaneta americana*. *Insect Biochem. Mol. Biol.* **2004**, *34*, 511-521.
5. Troppmann, B.; Balfanz, S.; Krach, C.; Baumann, A.; Blenau, W. Characterization of an invertebrate-type dopamine receptor of the American cockroach, *Periplaneta americana*. *Int. J. Mol. Sci.* **2014**, *15*, 629-653.
6. Troppmann, B.; Balfanz, S.; Baumann, A.; Blenau, W. Inverse agonist and neutral antagonist actions of synthetic compounds at an insect 5-HT$_1$ receptor. *Br. J. Pharmacol.* **2010**, *159*, 1450-1562.
7. Blenau, W.; Balfanz, S.; Baumann, A. Amtyr1: characterization of a gene from honeybee (*Apis mellifera*) brain encoding a functional tyramine receptor. *J. Neurochem.* **2000**, *74*, 900-908.
8. Reim, T.; Balfanz, S.; Baumann, A.; Blenau, W.; Thamm, M.; Scheiner, R. AmTAR2: Functional characterization of a honeybee tyramine receptor stimulating adenylyl cyclase activity. *Insect Biochem. Mol. Biol.* **2017**, *80*, 91-100.
9. Grohmann, L.; Blenau, W.; Erber, J.; Ebert, P.R.; Strünker, T.; Baumann, A. Molecular and functional characterization of an octopamine receptor from honeybee (*Apis mellifera*) brain. *J. Neurochem.* **2003**, *86*, 725-735.
10. Blenau, W.; Wilms, J.A.; Balfanz, S.; Baumann, A. AmOctα2R: functional characterization of a honeybee octopamine receptor inhibiting adenylyl cyclase activity. *Int. J. Mol. Sci.* **2020**, *21*, 9334.
11. Balfanz, S.; Jordan, N.; Langenstück, T.; Breuer, J.; Bergmeier, V.; Baumann, A. Molecular, pharmacological, and signaling properties of octopamine receptors from honeybee (*Apis mellifera*) brain. *J. Neurochem.* **2014**, *129*, 284-296.
12. Blenau, W.; Erber, J.; Baumann, A. Characterization of a dopamine D$_1$ receptor from *Apis mellifera*: cloning, functional expression, pharmacology, and mRNA localization in the brain. *J. Neurochem.* **1998**, *70*, 15-23.
13. Humphries, M.A.; Mustard, J.A.; Hunter, S.J.; Mercer, A.; Ward, V.; Ebert, P.R. Invertebrate D$_2$ type dopamine receptor exhibits age-based plasticity of expression in the mushroom bodies of the honeybee brain. *J. Neurobiol.* **2003**, *55*, 315-330.
14. Mustard, J.A.; Blenau, W.; Hamilton, I.S.; Ward, V.K.; Ebert, P.R.; Mercer, A.R. Analysis of two D1-like dopamine receptors from the honey bee *Apis mellifera* reveals agonist-independent activity. *Brain Res. Mol. Brain Res.* **2003**, *113*, 67-77.

15. Beggs, K.T.; Hamilton, I.S.; Kurshan, P.T.; Mustard, J.A.; Mercer, A.R. Characterization of a D2-like dopamine receptor (AmDOP3) in honey bee, *Apis mellifera*. *Insect Biochem. Mol. Biol.* **2005**, *35*, 873-882.
16. Thamm, M.; Balfanz, S.; Scheiner, R.; Baumann, A.; Blenau, W. Characterization of the 5-HT_{1A} receptor of the honeybee (*Apis mellifera*) and involvement of serotonin in phototactic behavior. *Cell. Mol. Life Sci.* **2010**, *67*, 2467-2479.
17. Thamm, M.; Rolke, D.; Jordan, N.; Balfanz, S.; Schiffer, C.; Baumann, A.; Blenau, W. Function and distribution of 5-HT_2 receptors in the honeybee (*Apis mellifera*). *PLoS One* **2013**, *8*, e82407.
18. Schlenstedt, J.; Balfanz, S.; Baumann, A.; Blenau, W. Am5-HT_7: molecular and pharmacological characterization of the first serotonin receptor of the honeybee (*Apis mellifera*). *J. Neurochem.* **2006**, *98*, 1985-1998.
19. Saudou, F.; Amlaiky, N.; Plassat, J.L.; Borrelli, E.; Hen, R. Cloning and characterization of a *Drosophila* tyramine receptor. *EMBO J.* **1990**, *9*, 3611-3617.
20. Cazzamali, G.; Klaerke, D.A.; Grimmelikhuijzen, C.J. A new family of insect tyramine receptors. *Biochem. Biophys. Res. Commun.* **2005**, *338*, 1189-1196.
21. Bayliss, A.; Roselli, G.; Evans, P.D. A comparison of the signalling properties of two tyramine receptors from *Drosophila*. *J. Neurochem.* **2013**, *125*, 37-48.
22. Balfanz, S.; Strünker, T.; Frings, S.; Baumann, A. A family of octopamine receptors that specifically induce cyclic AMP production or Ca^{2+} release in *Drosophila melanogaster*. *J. Neurochem.* **2005**, *93*, 440-451.
23. Qi, Y.X.; Xu, G.; Gu, G.X.; Mao, F.; Ye, G.Y.; Liu, W.; Huang, J. A new *Drosophila* octopamine receptor responds to serotonin. *Insect Biochem. Mol. Biol.* **2017**, *90*, 61-70.
24. Maqueira, B.; Chatwin, H.; Evans, P.D. Identification and characterization of a novel family of *Drosophila* β-adrenergic-like octopamine G-protein coupled receptors. *J. Neurochem.* **2005**, *94*, 547-560.
25. Gotzes, F.; Balfanz, S.; Baumann, A. Primary structure and functional characterization of a *Drosophila* dopamine receptor with high homology to human $D_{1/5}$ receptors. *Receptors Channels* **1994**, *2*, 131-141.
26. Feng, G.; Hannan, F.; Reale, V.; Hon, Y.Y.; Kousky, C.T.; Evans, P.D.; Hall, L.M. Cloning and functional characterization of a novel dopamine receptor from *Drosophila melanogaster*. *J. Neurosci.* **1996**, *16*, 3925-3933.
27. Hearn, M.G.; Ren, Y.; McBride, E.W.; Reveillaud, I.; Beinborn, M.; Kopin, A.S. A *Drosophila* dopamine 2-like receptor: Molecular characterization and identification of multiple alternatively spliced variants. *Proc. Natl. Acad. Sci. U. S. A.* **2002**, *99*, 14554-14559.
28. Srivastava, D.P.; Yu, E.J.; Kennedy, K.; Chatwin, H.; Reale, V.; Hamon, M.; Smith, T.; Evans, P.D. Rapid, nongenomic responses to ecdysteroids and catecholamines mediated by a novel *Drosophila* G-protein-coupled receptor. J. *Neurosci.* **2005**, *25*, 6145-6155.
29. Saudou, F.; Boschert, U.; Amlaiky, N.; Plassat, J.L.; Hen, R. A family of *Drosophila* serotonin receptors with distinct intracellular signalling properties and expression patterns. *EMBO J.* **1992**, *11*, 7-17.
30. Colas, J.F.; Launay, J.M.; Kellermann, O.; Rosay, P.; Maroteaux, L. *Drosophila* 5-HT_2 serotonin receptor: coexpression with fushi-tarazu during segmentation. *Proc. Natl. Acad. Sci. U. S. A.* **1995**, *92*, 5441-5445.
31. Blenau, W.; Stöppler, D.; Balfanz, S.; Thamm, M.; Baumann, A. Dm5-HT_{2B}: pharmacological characterization of the fifth serotonin receptor subtype of *Drosophila melanogaster*. *Front. Syst. Neurosci.* **2017**, *11*, 28.
32. Witz, P.; Amlaiky, N.; Plassat, J.L.; Maroteaux, L.; Borrelli, E.; Hen, R. Cloning and characterization of a *Drosophila* serotonin receptor that activates adenylate cyclase. *Proc. Natl. Acad. Sci. U. S. A.* **1990**, *87*, 8940-8944.
33. Simakov, O.; Kawashima, T.; Marlétaz, F.; Jenkins, J.; Koyanagi, R.; Mitros, T.; Hisata, K.; Bredeson, J.; Shoguchi, E.; Gyoja, F.; Yue, J.X.; Chen, Y.C.; Freeman, R.M. Jr.; Sasaki, A.; Hikosaka-Katayama, T.; Sato, A.; Fujie, M.; Baughman, K.W.; Levine, J.; Gonzalez, P.; Cameron, C.; Fritzenwanker, J.H.; Pani, A.M.; Goto, H.; Kanda, M.; Arakaki, N.; Yamasaki, S.; Qu, J.; Cree, A.; Ding, Y.; Dinh, H.H.; Dugan, S.; Holder, M.; Jhangiani, S.N.; Kovar, C.L.; Lee, S.L.; Lewis, L.R.; Morton, D.; Nazareth, L.V.; Okwuonu, G.; Santibanez, J., Chen, R., Richards, S., Muzny, D.M., Gillis, A.; Peshkin, L.; Wu, M.; Humphreys, T.; Su, Y.H.; Putnam, N.H.; Schmutz, J.; Fujiyama, A.; Yu, J.K.; Tagawa, K.; Worley, K.C.; Gibbs, R.A.; Kirschner, M.W.; Lowe, C.J.; Satoh, N.; Rokhsar, D.S.; Gerhart, J. Hemichordate genomes and deuterostome origins. *Nature* **2015**, *527*, 459-465.
34. Schwinn, D.A.; Lomasney, J.W.; Lorenz, W.; Szklut, P.J.; Fremeau, R.T. Jr.; Yang-Feng, T.L.; Caron, M.G.; Lefkowitz, R.J.; Cotecchia, S. Molecular cloning and expression of the cDNA for a novel α_1-adrenergic receptor subtype. *J. Biol. Chem.* **1990**, *265*, 8183-8189.
35. Allen, L.F.; Lefkowitz, R.J.; Caron, M.G.; Cotecchia, S. G-protein-coupled receptor genes as protooncogenes: constitutively activating mutation of the α_{1B}-adrenergic receptor enhances mitogenesis and tumorigenicity. *Proc. Natl. Acad. Sci. U. S. A.* **1991**, *88*, 11354-11358.
36. Bruno, J.F.; Whittaker, J.; Song, J.F.; Berelowitz. M. Molecular cloning and sequencing of a cDNA encoding a human α_{1A} adrenergic receptor. *Biochem. Biophys. Res. Commun.* **1991**, *179*, 1485-1490.
37. Kobilka, B.K.; Matsui, H.; Kobilka, T.S.; Yang-Feng, T.L.; Francke, U.; Caron, M.G.; Lefkowitz, R.J.; Regan, J.W. Cloning, sequencing, and expression of the gene coding for the human platelet α_2-adrenergic receptor. *Science* **1987**, *238*, 650-656.

38. Lomasney, J.W.; Lorenz, W.; Allen, L.F.; King, K.; Regan, J.W.; Yang-Feng, T.L.; Caron, M.G.; Lefkowitz, R.J. Expansion of the α_2-adrenergic receptor family: cloning and characterization of a human α_2-adrenergic receptor subtype, the gene for which is located on chromosome 2. *Proc. Natl. Acad. Sci. U. S. A.* **1990**, *87*, 5094-5098.
39. Regan, J.W.; Kobilka, T.S.; Yang-Feng, T.L.; Caron, M.G.; Lefkowitz, R.J.; Kobilka, B.K. Cloning and expression of a human kidney cDNA for an α_2-adrenergic receptor subtype. *Proc. Natl. Acad. Sci. U. S. A.* **1988**, *85*, 6301-6305.
40. Frielle, T.; Collins, S.; Daniel, K.W.; Caron, M.G.; Lefkowitz, R.J.; Kobilka, B. K. Cloning of the cDNA for the human β_1-adrenergic receptor. *Proc. Natl. Acad. Sci. U. S. A.* **1987**, *84*, 7920-7924.
41. Emorine, L.J.; Marullo, S.; Delavier-Klutchko, C.; Kaveri, S.V.; Durieu-Trautmann, O.; Strosberg, A.D. Structure of the gene for human β_2-adrenergic receptor: expression and promoter characterization. *Proc. Natl. Acad. Sci. U. S. A.* **1987**, *84*, 6995-6999.
42. Emorine, L.J.; Marullo, S.; Briend-Sutren, M.M.; Patey, G.; Tate, K.; Delavier-Klutchko, C.; Strosberg, A.D. Molecular characterization of the human β_3-adrenergic receptor. *Science* **1989**, *245*, 1118-1121.
43. Sunahara, R.K.; Niznik, H.B.; Weiner, D.M.; Stormann, T.M.; Brann, M.R.; Kennedy, J.L.; Gelernter, J.E.; Rozmahel, R.; Yang, Y.L.; Israel, Y.; Seeman, P.; O'Dowd, B.F. Human dopamine D_1 receptor encoded by an intronless gene on chromosome 5. *Nature* **1990**, *347*, 80-83.
44. Sunahara, R.K.; Guan, H.C.; O'Dowd, B.F.; Seeman, P.; Laurier, L.G.; Ng, G.; George, S.R.; Torchia, J.; Van Tol, H.H.; Niznik, H.B. Cloning of the gene for a human dopamine D_5 receptor with higher affinity for dopamine than D_1. *Nature* **1991**, *350*, 614-619.
45. Dearry, A.; Falardeau, P.; Shores, C.; Caron, M.G. D_2 dopamine receptors in the human retina: cloning of cDNA and localization of mRNA. *Cell. Mol. Neurobiol.* **1991**, *11*, 437-453.
46. Sokoloff, P.; Giros, B.; Martres, M.P.; Bouthenet, M.L.; Schwartz J.C. Molecular cloning and characterization of a novel dopamine receptor (D_3) as a target for neuroleptics. *Nature* **1990**, *347*, 146-151.
47. Van Tol, H.H.; Bunzow, J.R.; Guan, H.C.; Sunahara, R.K.; Seeman, P.; Niznik, H.B., Civelli, O. Cloning of the gene for a human dopamine D_4 receptor with high affinity for the antipsychotic clozapine. *Nature* **1991**, *350*, 610-614.
48. Fargin, A.; Raymond, J.R.; Lohse, M.J.; Kobilka, B.K.; Caron, M.G.; Lefkowitz, R.J. The genomic clone G-21 which resembles a β-adrenergic receptor sequence encodes the 5-HT_{1A} receptor. *Nature* **1988**, *335*, 358-360.
49. Jin, H.; Oksenberg, D.; Ashkenazi, A.; Peroutka, S.J.; Duncan, A.M.; Rozmahel, R.; Yang, Y.; Mengod, G.; Palacios, J.M.; O'Dowd, B.F. Characterization of the human 5-$hydroxytryptamine_{1B}$ receptor. *J. Biol. Chem.* **1992**, *267*, 5735-5738.
50. Hamblin, M.W.; Metcalf, M.A. Primary structure and functional characterization of a human 5-HT_{1D}-type serotonin receptor. *Mol. Pharmacol.* **1991**, *40*, 143-148.
51. Levy, F.O.; Gudermann, T.; Perez-Reyes, E.; Birnbaumer, M.; Kaumann, A.J.; Birnbaumer, L. Molecular cloning of a human serotonin receptor (S12) with a pharmacological profile resembling that of the 5-HT_{1D} subtype. *J. Biol. Chem.* **1992**, *267*, 7553-7562.
52. Adham, N.; Kao, H.T.; Schecter, L.E.; Bard, J.; Olsen, M.; Urquhart. D.; Durkin, M.; Hartig, P.R.; Weinshank, R.L.; Branchek, T.A. Cloning of another human serotonin receptor (5-HT1F): a fifth 5-HT1 receptor subtype coupled to the inhibition of adenylate cyclase. *Proc. Natl. Acad. Sci. U. S. A.* **1993**, *90*, 408-412.
53. Saltzman, A.G.; Morse, B.; Whitman, M.M.; Ivanshchenko, Y.; Jaye, M.; Felder, S. Cloning of the human serotonin 5-HT_2 and 5-HT_{1C} receptor subtypes. *Biochem. Biophys. Res. Commun.* **1991**, *181*, 1469-1478.
54. Schmuck, K.; Ullmer, C.; Engels, P.; Lubbert, H. Cloning and functional characterization of the human 5-HT_{2B} serotonin receptor. *FEBS Lett.* **1994**, *342*, 85-90.
55. Lovenberg, T.W.; Baron, B.M.; de Lecea, L.; Miller, J.D.; Prosser, R.A.; Rea, M.A.; Foye, P.E.; Racke, M.; Slone, A.L.; Siegel, B.W.; Danielson, P.A.; Sutcliffe, J.G.; Erlander M.G. A novel adenylyl cyclase-activating serotonin receptor (5-HT_7) implicated in the regulation of mammalian circadian rhythms. *Neuron* **1993**, *11*, 449-458.
56. Nathans, J.; Hogness, D.S. Isolation and nucleotide sequence of the gene encoding human rhodopsin. *Proc. Natl. Acad. Sci. U. S. A.* **1984**, *81*, 4851-4855.
